# miRNAs in Extracellular Vesicles from iPS-Derived Cardiac Progenitor Cells Effectively Reduce Fibrosis and Promote Angiogenesis in Infarcted Heart

**DOI:** 10.1155/2019/3726392

**Published:** 2019-11-11

**Authors:** Wanling Xuan, Lei Wang, Meifeng Xu, Neal L. Weintraub, Muhammad Ashraf

**Affiliations:** ^1^Vascular Biology Center, Medical College of Georgia at Augusta University, Augusta, Georgia, USA; ^2^Department of Pharmacology, University of Illinois at Chicago College of Medicine, Chicago, Illinois, USA; ^3^Department of Pathology and Laboratory Medicine, University of Cincinnati Medical Center, Cincinnati, Ohio, USA

## Abstract

Cardiac stem cell therapy offers the potential to ameliorate postinfarction remodeling and development of heart failure but requires optimization of cell-based approaches. Cardiac progenitor cells (CPCs) induction by ISX-9, a small molecule possessing antioxidant, prosurvival, and regenerative properties, represents an attractive potential approach for cell-based cardiac regenerative therapy. Here, we report that extracellular vesicles (EV) secreted by ISX-9-induced CPCs (EV-CPC^ISX-9^) faithfully recapitulate the beneficial effects of their parent CPCs with regard to postinfarction remodeling. These EV contain a distinct repertoire of biologically active miRNAs that promoted angiogenesis and proliferation of cardiomyocytes while ameliorating fibrosis in the infarcted heart. Amongst the highly enriched miRNAs, miR-373 was strongly antifibrotic, targeting 2 key fibrogenic genes, GDF-11 and ROCK-2. miR-373 mimic itself was highly efficacious in preventing scar formation in the infarcted myocardium. Together, these novel findings have important implications with regard to prevention of postinfarction remodeling.

## 1. Introduction

Myocardial infarction (MI) and subsequent heart failure are a leading cause of death worldwide [1]. Despite advances in medical and device therapies, heart failure continues to be associated with a 5-year mortality of ~50%. Stem cell therapy thus offers a great potential for cardiac tissue repair and regeneration, which might ultimately improve symptoms and longevity [2].

Notably, the beneficial effects of cardiac stem cell therapy are largely attributed to a paracrine mechanism of action that involves the release of cellular factors from the transplanted stem cells [3–5]. More recent studies show that these factors are packed into small membrane-bound vesicles known as extracellular vesicles (EV, 30-200 nm), which can invoke a multitude of signals [6, 7]. The EV contents vary amongst stem cells. Cardiac progenitor cells (CPCs) are of particular interest due to their inherent properties of cell protection, cell development, differentiation, and desirable effects imparted into the host tissue [8–10]. EV from newborns improved ventricular remodeling post-MI significantly more than those derived from aging patients [11]. Similarly, EV secreted from young cardiosphere-derived cells exerted stronger antisenescent effects than those derived from aged animals [12].

Recent studies demonstrated that effects of CPCs on cardiac repair and regeneration can be faithfully recapitulated by their EV [6, 13]. Multiple miRNAs in EV act as mediators of cell-cell communication within the cardiovascular system [2] and can be transferred into recipient cells to regulate gene expression, thus leading to cardioprotection [11, 13, 14]. We reported that a small molecule, ISX-9, could render CPCs (CPC^ISX-9^) highly resistant to oxidative stress, thus permitting better survival and engraftment in the infarcted myocardium [15]. Development of CPC^ISX-9^ may represent a significant advance in the cardiac stem cell field, as ISX-9 treatment circumvents the need to genetically reprogram the cells in order to enhance their function. Since CPC^ISX-9^ are well positioned for therapeutic application in humans, characterizing EV secreted from these cells is not only important to provide insight into their mechanisms of action, but may also help to identify novel miRNAs involved in cardioprotection.

Since the EV cargo contents are unique to each cell type, consequently, their effectiveness is variable. Considering this limitation, we have generated multipotent CPCs from human-induced pluripotent stem cells (hiPSCs) using a unique small molecule with antioxidant and regenerative properties capable of successfully propagating in the infarcted myocardium. Since CPCs are the cells of choice for regeneration, their EV would be considered to be more effective in cardiac repair than EV from non-CPC. Therefore, the purpose of the study was to exploit EV from hiPSC-CPC induced with ISX-9 and not the role of ISX-9 per se on EV release from CPC. Here, we tested the hypothesis that EV secreted by ISX-9-induced CPCs (EV-CPC^ISX-9^) will be highly efficacious in cardiac repair owing to the unique properties of their parent cells. EV-CPC^ISX-9^ exerted strong effects on fibrosis and angiogenesis in the infarcted myocardium of mice. Mechanistically, we identified miR-373 enriched in EV-CPC^ISX-9^, which elicited strong antifibrotic effects by targeting two genes, growth differentiation factor 11 (GDF-11) and Rho-associated coiled-coil containing kinase-2 (ROCK-2), and showed that the miR-373 mimic effectively inhibits postinfarct cardiac remodeling.

## 2. Materials and Methods

### 2.1. Cell Culture

Human iPSC cell line (ACS-1021, ATCC, USA) was maintained in mTeSR1 media (Stem Cell Technology) on vitronectin-coated six-well plates with daily medium changes. Cells were passaged with ReLeSR™ reagent every 4-7 days according to the manufacturer's protocol (Stem Cell Technology). For CPC generation, briefly, hiPSCs maintained on vitronectin-coated six-well plates in mTeSR1 media (Stem Cell Technology) were dissociated into single cells using Accutase (Invitrogen) at 37°C for 10 min and then were seeded on to vitronectin-coated six-well plates at 1 × 10^6^ cells/well in mTeSR1 supplemented with 5 *μ*M ROCK inhibitor (Y-27632, Stem Cell Technology) for 24 h. The following day, cells were cultured in mTesR1 with daily medium change for 3 days. Afterwards, the medium was switched to RPMI/B27 minus insulin supplemented with ISX-9 (20 *μ*M, dissolved in DMSO, Stem Cell Technology) for 7 days. Embryoid bodies (EBs) were generated using the hanging drop method in RPMI/B27 minus insulin medium. Human dermal fibroblast cell line (CC-2511) and lung fibroblast cell line (CC-2512) were obtained from the Lonza Company. Briefly, fibroblasts were maintained in FibroGRO™ Complete Media (Millipore Sigma). Cells were passaged with Accutase; passages 2-4 were used for experiments. Commercial human CPCs (control-CPC) derived from human iPS cells (catalog: R1093, Cellular Dynamics International) were maintained in serum-free William's E Medium supplemented with Cocktail B (CM400, Life Technologies). Passage 2 was used for experiments.

### 2.2. Isolation of EV

Human iPSC cell line ACS-1021 (ATCC, USA) and CPCs induced by ISX-9 were cultured as described [15]. In some cases, EBs and commercial human CPCs were also cultured. Conditioned media was collected and EVs were isolated by centrifugation at 3000 rpm for 30 min to remove cells and debris, followed by filtration through a 0.22 *μ*m filter to remove the remaining debris. Then, the medium was further concentrated to 500 *μ*l using Amicon Ultra-15 100 kDa centrifugal filter units (Millipore). Isolation of EV in the concentrated medium was carried out through qEV size exclusion columns (Izon Science). EV fractions were collected and concentrated by Amicon Ultra-4 10 kDa centrifugal filter units to a final volume of <100 *μ*l. The purified EVs were stored at -80°C and subsequently characterized by particle size, EV markers, and electron microscopy.

### 2.3. Particle Size and Concentration Distribution Measurement with Tunable Resistive Pulse Sensing

Particle size and concentration distribution were performed using tunable resistive pulse sensing (TRPS) technique with a qNano instrument (Izon Science). Briefly, the number of particles were counted (at least 600 to 1000 events) using 20 mbar pressure and NP200 nanopore membranes stretched between 46.5 and 47.5 mm. Calibration was performed using known concentration of CPC200 beads (diameter: 210 nm). Data were processed using Izon Control Suite software.

### 2.4. Transmission Electron Microscopy

EV pellets were fixed with 4% paraformaldehyde (PFA). Following a total of 8 washes using PBS, grids were contrasted with a uranyl-oxalate solution for 5 minutes and transferred to methyl-cellulose-uranyl acetate for 10 minutes on ice as previously described [16]. Samples were examined on a JEOL JEM-1220 transmission electron microscope (TEM) (JEOL USA, Inc.).

### 2.5. EV Uptake by Fibroblasts

To track EV uptake by cultured fibroblasts, purified EV were labeled with PKH26, a red membrane dye (Sigma-Aldrich), according to the manufacturer's protocol. Briefly, 300 *μ*l of EV was suspended into 100 *μ*l of Diluent C, which was mixed with 1.4 *μ*l of PKH26 dye. The labeling reaction was stopped by adding an equal volume of EV-free FBS. Exosome Spin Columns (Cat. 4484449, Thermo Fisher Scientific) were used to remove unincorporated PKH26. The cultured fibroblasts in the slide chamber were incubated with labeled EV at 37°C for 24 h. After incubation, cells were stained with Calcein AM (5 *μ*M). Cells were fixed with 2% formaldehyde for 5 min and mounted with DAPI containing ProLong Gold Antifade medium (Thermo Fisher Scientific). Images were taken with an FV1000 confocal microscope (Olympus, Japan).

### 2.6. Cell Transfection and In Vitro Fibrosis Assay

Experiments were performed using CPC^ISX-9^ grown in RPMI/B27 minus insulin, 25 nM miR-373 mimic, anti-miR-373, negative controls, and RNAiMAX (Invitrogen) according to the manufacturer's instructions. miR-373 mimic and anti-miR-373 (inhibitor) were synthesized by Ambion (Life Technologies). The sequence of the miR-373 inhibitor was as follows: anti-miR-373, 5′-ACACCCCAAAAUCGAAGCACUUC-3′. miRNA mimic negative control (#4464066, Ambion) and miRNA inhibitor negative control (#4464076, Ambion) were obtained from the Life Technologies company. After 24 h transfection, cells remained in culture for 24 h and EVs from different cell groups were collected for experimentation. The transfection efficiency was analyzed using real-time PCR. In order to test the antifibrotic potential of miR-373 from EV-CPC^ISX-9^, fibroblasts were cocultured with EV (1∗10^8^/ml) from anti-miR373 inhibitor-treated CPC^ISX-9^, negative control-treated CPC^ISX-9^, or miR-373 mimic for 48 h, and then fibroblasts were grown in serum-free DMEM medium with or without TGF-*β* (10 ng/ml, R&D) for 48 h. Expression of profibrotic genes was analyzed by real-time PCR. For the hypoxia assay, lung fibroblasts and dermal fibroblasts in culture were randomly divided into five groups and treated with miR-NC, anti-miR, miR-373 mimic, EV-CPC^ISX-9^, and EV-CPC^ISX-9^+anti-miR-373. After 24 h of different pretreatments, cells were subjected to 1% O_2_ in a hypoxic chamber (InvivO_2_ 500) for 72 h. Then, cells were fixed with 4% formaldehyde for 10 mins and stained with *α*-SMA (ab5694, Abcam, 1 : 200). Signals were visualized with Alexa Fluor 488 secondary antibodies (Life Technologies).

### 2.7. miRNA Array Analysis

The NanoString nCounter Human v3 miRNA Expression Assay was used to perform the microRNA profiling analysis. The assay allows measurement of 800 different microRNAs at the same time for each sample. 3.5 *μ*l of suspension RNA was annealed with multiplexed DNA tags (miR-tag) and bridges target specifics. Mature microRNAs were bonded to specific miR-tags using a ligase enzyme, and excess tags were removed by enzyme clean-up step. The tagged microRNA product was diluted 1 to 5, and 5 *μ*l was combined with 20 *μ*l of reporter probes in a hybridization buffer and 5 *μ*l of capture probes overnight (17 hours) at 65°C to permit hybridization of probes with specific target sequences. Excess probes were removed using two-step magnetic bead-based purification on an automated fluidic handling system (nCounter Prep Station), and target/probe complexes were immobilized on the cartridge for data collection. The nCounter Digital Analyzer took images of immobilized fluorescent reporters in the sample cartridge with a CCD camera through a microscope objective lens. For each cartridge, a high-density scan encompassing 325 fields of view was performed. NanoString raw data was analyzed with nSolver™ software, provided by NanoString Technologies. The mean plus 2 times the standard deviation of negative control probes was used to perform background subtraction; positives were used to perform technical normalization to adjust lane by lane variability due to differences in hybridization, purification, or binding. Data was then normalized by calculating the geometric mean of the spikes present in each sample, as recommended by NanoString. One-way ANOVA was used to calculate the *P* value; targets with *P* < 0.05 were selected.

### 2.8. miRNA Target Gene Prediction, Gene Ontology (GO) Analysis, and Luciferase Activity Assay

miRNA target gene prediction and gene ontology analysis were carried out using DIANA MR-microT and mirPath software. Differential miRNA target genes in significant GO and pathway categories, obtained from GO and pathway analyses, were analyzed with mirPath v.3 software. GO biological process includes biological processes, molecular function, and cellular component of upregulated and downregulated genes.

**Figure 1:**
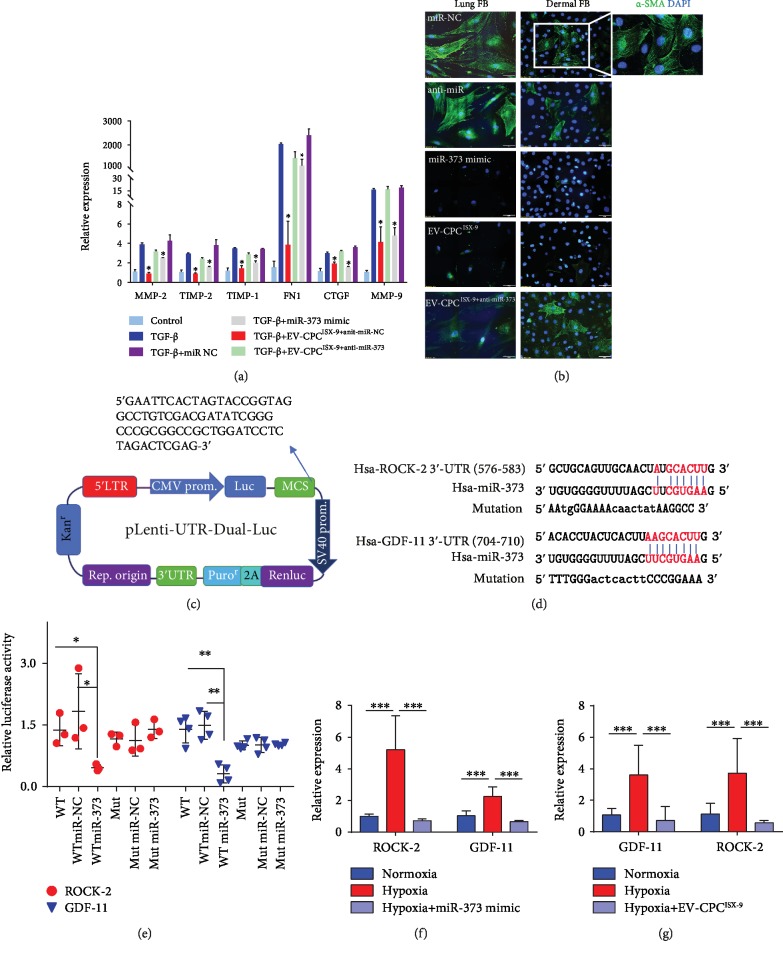
Fibrotic gene expression in fibroblasts after TGF-*β* stimulation. (a) Effects of EV-CPC^ISX-9^ on fibrotic gene expression: role of miR-373. *n* = 6. (b) Transdifferentiation of lung fibroblasts and dermal fibroblasts into myofibroblasts by hypoxia for 72 h as detected by immunostaining for *α*-smooth actin (*α*-SMA): effects of EV-CPC^ISX-9^ and miR-373 mimic pretreatment. Bar = 50 *μ*m. (c) Schematic representation of the luciferase reporter constructs. (d) Sequence alignment of miR-373 with the human wild-type (WT) ROCK-2 3′-UTR and GDF-11 3′-UTR and mutated reporters. The seed sequence (red) is highlighted. (e) Relative luciferase activity (relative, firefly luciferase activity/Renilla luciferase activity) of 293FT cells cotransfected with WT 3′-UTR-ROCK-2 or GDF-11 and mutant 3′-UTR-ROCK-2 or GDF-11 and miR-373 mimics vs. NC. ^∗∗^*P* < 0.01, *n* = 4. UTR: untranslated region; miRNA: microRNA; NC: negative control; WT: wild type. (f) 72 h hypoxia increased GDF-11 and ROCK-2 mRNA expression in lung fibroblasts: effects of pretreatment with miR-373 mimic. ^∗∗∗^*P* < 0.001. n = 6. (g) Pretreatment of fibroblasts during hypoxia with EV-CPC^ISX-9^ significantly decreased the upregulation of GDF-11 and ROCK-2 similar to pretreatment with miR-373 mimic. ^∗∗∗^*P* < 0.001. *n* = 6.

For luciferase activity assay, using standard procedures, wild-type (WT), or mutant 3′untraslated regions (UTRs) of GDF-11 or ROCK-2 were subcloned into the pLenti-UTR-Dual-Luc vector (abm, Canada) (Figure 1(c)) downstream of the luciferase gene. The predicted binding sites and mutant sequences are shown in Figure 1(d). GDF-11-3′-UTR-WT, GDF-11-3′-UTR-Mut, ROCK2-3′-UTR-WT, or ROCK2-3′-UTR-Mut vectors were cotransfected with miR-373 mimic or negative control into 293FT cells using Lipofectamine 3000 for 48 h. Transfected cells were analyzed using the dual-luciferase reporter assay system (Promega). The luciferase activity was normalized using Renilla activity.

### 2.9. Myocardial Infarction Model

Animal experiments were carried out both at the University of Illinois at Chicago and Augusta University according to experimental protocols approved by the University of Illinois at Chicago and Augusta University Animal Care and Use Committee, and the methods were performed in accordance with the guide for the Care and Use of Laboratory Animals by the Institute of Animal Resources. MI model was generated as previously described [15]. Briefly, MI was induced in 8-9-week-old NOD/SCID mice (The Jackson Laboratory) or C57/B6 mice which were anaesthetized with 2% isoflurane (isoflurane USP, Henry Schein), intubated, and ventilated. The left anterior descending (LAD) coronary artery was permanently ligated with a Prolene #8-0 suture. 10 mins after LAD ligation, EV (1∗10^12^/ml) from hiPSC or CPC^ISX-9^ were injected into the myocardium along the border zone with a total of 20 *μ*l. The same volume of PBS was injected in the control group. miR-373 mimic in vivo transfection was performed in C57/B6 mice. in vivo-jetPEI™ system (Polyplus-transfection SA) was used for intracardiac miRNA delivery. 200 pmoles of miR-373 mimic complexed with in vivo-jetPEI at a N/P ratio of 7 in a volume of 20 *μ*l was injected into the myocardium along the border zone approximately 10 mins after LAD ligation.

### 2.10. Echocardiography

Echocardiography was performed in mice anesthetized mildly with inhaled isoflurane (0.5%) using a Philips iE33 ultrasound machine, equipped with a L15-7io probe as described previously [15]. Hearts were imaged in 2D in the parasternal long-axis and/or parasternal short-axis views at the level of the highest LV diameter. Measurements of left ventricular end diastolic diameter (LVDd) and left ventricular end systolic diameter (LVDs) were made from 2D M-mode images of the left ventricle in both the systole and diastole. Left ventricle fractional shortening (LVFS) was calculated using the following formula: LVFS = (LVDd − LVDs)/LVDd × 100. Ejection fraction (EF), left ventricular end diastolic volume (LVEDv), and left ventricular end systolic volume (LVESv) were calculated using the following formula: 7.0 × LVEDd^3^/(2.4 + LVDd) and 7.0 × LVESd^3^/(2.4 + LVDs), respectively; left ventricular ejection fraction (LVEF) was calculated as (LVEDv − LVESv)/LVEDv × 100%. LVFS and EF were expressed as percentages.

### 2.11. Histology

Histological analysis was performed in randomly selected hearts from mice subjected to MI and treatment groups (PBS, EV-hiPSC, or EV-CPC^ISX-9^ (*n* = 3 per group)). Mice were sacrificed after 1 month of treatment with EV. For immunostaining, hearts were fixed with 4% PFA for 1 hour at room temperature and replaced by 30% sucrose overnight at 4°C. Afterwards, hearts were cryopreserved in an optical cutting temperature (OCT) compound (Tissue-Tek) at -80°C. Hearts were sliced into 5 *μ*m thick frozen sections and incubated with primary antibodies including *α*-sarcomeric actinin (A7811, Sigma, 1 : 200), Ki67 (ab16667, Abcam, 1 : 500), cTnT (13-11, Thermo fisher Scientific, 1 : 300), and SMA (ab5694, Abcam, 1 : 300). Signals were visualized with Alexa Fluor 647 and Alexa Fluor 488 secondary antibodies (Life Technologies). Images were recorded on a confocal microscope (FV1000, Olympus, Japan). For fibrosis analysis, hearts were embedded in paraffin and cut at 5 *μ*m thick sections. Masson's trichrome staining was performed according to the manufacturer's protocol (HT-15, Sigma). The size of the LV area and the scar area was measured using the ImageJ software. 4 sections (EV-treated mice) and 6 sections (miR-373 mimic-treated mice) were analyzed per heart. The fibrosis area was determined as the ratio of the scar area to the LV area and expressed as a percentage. Vessel density was assessed in 9 animals (3 in each group) in NOD/SCID mice and 6 animals in C57/B6 mice (3 in each group) which were sacrificed at 1 M after MI. The number of vessels was blindly counted on 27 sections (3 sections per heart) in NOD/SCID mice or 18 sections (3 sections per heart) in C57/B6 mice in the infarct and border areas of all mice after staining with an antibody *α*-SMA using a fluorescence microscope at a 400x magnification. Vascular density was determined by counting *α*-SMA-positive vascular structures. The number of vessels in each section was averaged and expressed as the number of vessels per field (0.2 mm^2^).

### 2.12. Western Blot

EV and cell extracts were lysed with radio immunoprecipitation assay (RIPA) buffer supplemented with Complete Protease Inhibitor Mixture tablets (Roche Diagnostics). Protein concentration was determined by the Pierce™ BCA Protein Assay Kit (Thermo Scientific). 10 *μ*g proteins were separated by SDS/PAGE and transferred to the PVDF membrane (Bio-Rad). Membranes were incubated with primary antibodies against the following proteins overnight at 4°C: mouse anti-Tsg101 (sc-365062, Santa Cruz), mouse anti-calnexin (sc-23954, Santa Cruz), goat-anti-Hsp70 (EXOAB-Hsp70A-1, SBI), rabbit anti-CD9 (#13174, CST), rabbit anti-flotillin-1 (#18634, CST), and mouse anti-GADPH (sc-365062, Santa Cruz). The membrane was then washed and incubated with an anti-mouse/rabbit/goat peroxidase-conjugated secondary antibody. Immunoreactive bands were visualized by the enhanced chemiluminescence method (Pierce, Thermo Scientific) with a western blotting detection system (FluorChem E, ProteinSimple USA) and were quantified by densitometry with ImageJ software.

### 2.13. RNA Extraction and Real-Time PCR

Total RNA from EV was isolated using miRNeasy Micro Kit (QIAGEN). Reverse transcription was performed using a miScript II RT Kit (QIAGEN). Quantification of mRNA and selected miRNAs was performed by the real-time system QuantStudio 3 (ABI) using a miScript SYBR Green PCR Kit (QIAGEN). miRNA primer sequences are shown in [Supplementary-material supplementary-material-1], and mRNA primer sequences are shown in [Supplementary-material supplementary-material-1]. Expression levels of selected miRNAs were quantified and validated with RT-PCR, and values are expressed as 2^-*ΔΔ*CT^ with respect to the expression of the reference U6. The primer of U6 was provided in the PCR kit.

### 2.14. Statistical Analysis

Data are expressed as mean ± SD. Test for normality of data was performed. Statistical analysis of differences was compared by ANOVA with Bonferroni's correction for multiple comparisons. Comparisons between two groups were evaluated with Student's *t*-test. A probability value of *P* < 0.05 was considered statistically significant. Statistical analyses were performed using GraphPad Prism 6.0 (Chicago, USA).

## 3. Results

### 3.1. Characterization of EV Secreted by ISX-9-Induced Cardiac Progenitors

**Figure 2:**
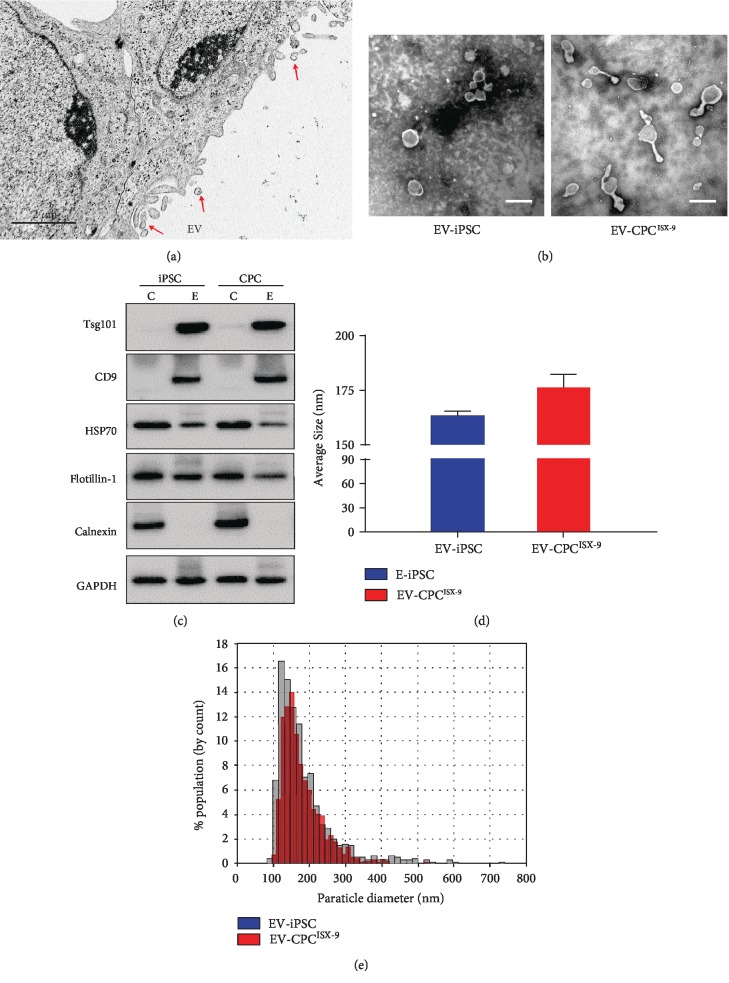
Characterization of EV from iPSC and CPC^ISX-9^. (a) Secretion of EV from the CPC^ISX-9^ as imaged by electron microscopy. Inset shows higher magnification of secreted EV (small black arrows). Blue arrows point to EV exiting from the cells. Bar = 1 *μ*m. (b) EV isolated from iPSC and CPC^ISX-9^ visualized by transmission electron microscopy (TEM). Scale bar = 200 nm. (c) Representative images of western blot for Tsg101, CD9, Hsp70, flotillin-1, and calnexin in EV lysates. C: cell lysate; E: EV. (d) Average size of EV as measured by TRPS. No significant difference in average size of EV from iPSC and CPC^ISX-9^ was observed. (e) Representative graph of size distribution of EV from iPSC and CPC^ISX-9^ as detected by TRPS.

Electron microscopy analysis showed that secreted EV measured 160-170 nm in diameter (Figures 2(a), 2(b), and 2(d)). No significant difference in size was observed amongst the groups (Figures 2(d) and 2(e)). Additionally, EV were enriched in EV-specific markers Tsg101, CD9, Hsp70, and flotillin-1. Calnexin was absent in isolated EV (Figure 2(c)), confirming their purity.

### 3.2. EV-CPC^ISX-9^ Exhibit a Unique miRNA Profile

**Figure 3:**
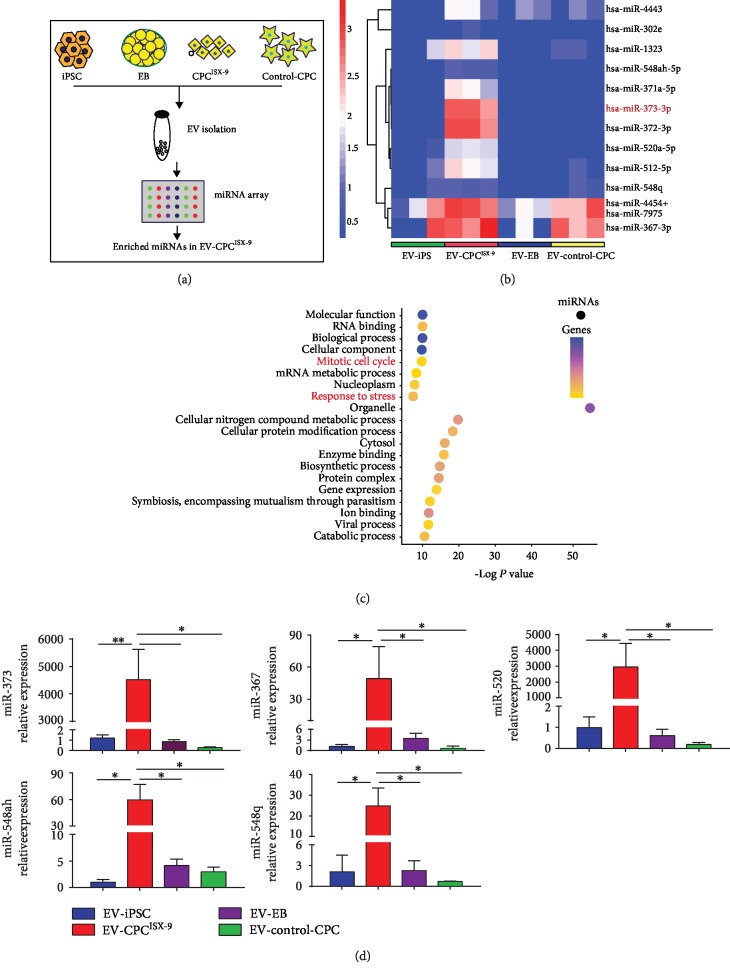
miRNA expression profiling and validation of microarray data. (a) Outline of experimental procedure. (b) Heatmap analysis of microarray data showing significant upregulation of miRNAs in EV-CPC^ISX-9^ compared with EV-iPSC, EV-EB, or EV-control-CPC. Red or blue colors indicate differentially up- or downregulated miRNA, respectively (*P* < 0.05). *n* = 3. (c) Biological process of Gene Ontology (GO) enrichment analysis based on miRNA-targeted genes. GO enrichment was analyzed with mirPath v.3 software. GO biological process includes biological processes, molecular function, and cellular component of upregulated and downregulated genes. (d) Validation of microarray data using real-time PCR. Quantitative results showing significant expression of miR-373, miR-367, miR-520, miR-548ah, and miR-548q in EV-CPC^ISX-9^. RNA samples were from three individual experiments. ^∗^*P* < 0.001.

Next, we performed a miRNA array to determine whether miRNA cargo content of EV-CPC^ISX-9^ differs from that of hiPSCs, EBs, and commercial CPCs (Figure 3(a)). Global miRNA profiling showed that EV-CPC^ISX-9^ had a unique miRNA expression signature very different from that of the other derived EVs. miR-520/-373 family members, including miR-371, miR-302, miR-372, miR-373, and miR-520, as well as miR-512, miR-548, and miR-367, were significantly upregulated in EV-CPC^ISX-9^ compared to EV from other parent cells (Figure 3(b)) (GSE126347). Furthermore, the expression of these enriched miRNAs (miR-373/miR-548/miR-367/miR-520) was validated with real-time PCR (Figure 3(d)). The target genes of the differentially expressed miRNAs control a broad range of biological functions. The biological process of Gene Ontology (GO) enrichment analysis based on enriched miRNA-targeted genes demonstrated that some target genes were significantly enriched in responses to stress and cell cycle (Figure 3(c)).

### 3.3. Antifibrotic Effects Mediated by miR-373 Derived from EV-CPC^ISX-9^

We hypothesized that the enriched miR-373 EVs from CPC^ISX-9^ exert antifibrotic effects. First, using PKH26 labeling, we confirmed that EV were internalized by fibroblasts and localized in the perinuclear region ([Supplementary-material supplementary-material-1]). The miR-373 expression level was markedly higher in EV than in their donor cells, CPC^ISX-9^ ([Supplementary-material supplementary-material-1]). Inhibition of miR-373 in CPC^ISX-9^ resulted in decreased miR-373 expression in EV-CPC^ISX-9^ ([Supplementary-material supplementary-material-1]), and reduced miR-373 expression in fibroblasts incubated with these EV compared to those from control cells ([Supplementary-material supplementary-material-1]). Stimulation of fibroblasts with TGF-*β* led to significant upregulation of fibrotic genes (MMP-2, TIMP-2, TIMP-1, FN1, CTGF, and MMP-9). Upon pretreatment of fibroblasts with EV from control CPC^ISX-9^, upregulation of these fibrotic genes by TGF-*β* was inhibited. However, inhibition of miR-373 in CPC^ISX-9^ abrogated the capacity of the EV to inhibit fibrotic gene expression. Conversely, pretreatment of fibroblasts with miR-373 mimic inhibited TGF-*β*-induced expression of fibrotic genes (Figure 1(a)). We also performed experiments in a second cellular model of fibrosis by exposing the fibroblasts to hypoxia.  Figure 1(b) shows that with 72 h exposure in a hypoxic environment, both lung and dermal fibroblasts differentiated into myofibroblasts expressing *α*-SMA. As expected, the miR-373 mimic significantly inhibited fibroblast transdifferentiation into myofibroblasts under hypoxia. Similarly, fibroblasts failed to transdifferentiate into myofibroblasts when they were pretreated with EV from CPC^ISX-9^. Taken together, these results suggest that the miR-373 contained in EV-CPC^ISX-9^ suppresses fibrosis both in *in vitro* and *in vivo* levels.

Although a previous study reported miR-373 might target TGF-*β* [17], here we identified two new potential target genes of miR-373, GDF-11 and ROCK-2, using DIANA MR-microT software and dual-luciferase reporter assay. The 3′-UTR binding sites are shown in Figure 1(d). Next, the predicted binding sites of GDF-11 and ROCK-2 were cloned into 3′-UTR of the dual-luciferase vector (Figure 1(c)) and transiently transfected into 293FT cells. The miR-373 mimic transfection significantly decreased the relative luciferase activity when cotransfected with GDF-11 and ROCK-2 3′-UTR vectors. When 3′-UTR binding sites were mutated, the repression of GDF-11 and ROCK-2 3′-UTR by miR-373 mimic was attenuated (Figure 1(e)). Notably, the human miR-373 3′-UTR binding sites for GDF-11 and ROCK-2 are conserved among several species, including mice and rats (Figures [Supplementary-material supplementary-material-1]).

Moreover, under hypoxic conditions, the expression of GDF-11 and ROCK-2 was increased in lung fibroblasts (Figure 1(f)), while pretreatment with the miR-373 mimic or EV-CPC^ISX-9^ significantly inhibited expression of these two genes, supporting the contention that miR-373 targets GDF-11 and ROCK-2 (Figure 1(g)).

### 3.4. EV-CPC^ISX-9^ Promoted CM Proliferation and Angiogenesis and Reversed Ventricular Remodeling in Mice Post MI

**Figure 4:**
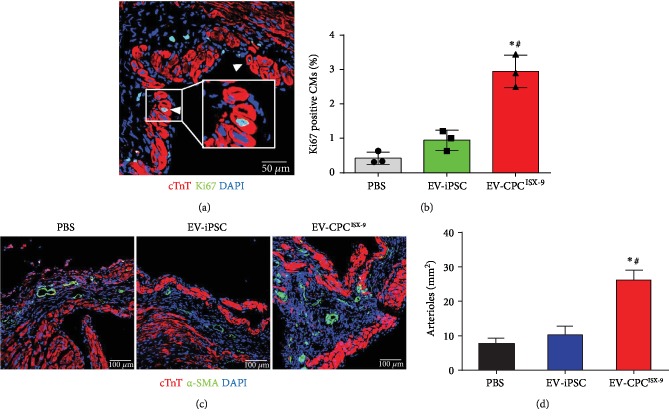
CPC^ISX-9^-derived EV promoted cardiomyocyte proliferation and angiogenesis after myocardial infarction (MI) in mice. (a) Representative image of Ki67-positive cardiomyocytes (cTnT positive) in EV-CPC^ISX-9^-treated mouse hearts 30 days after MI. Bar = 50 *μ*m. (b) Quantitative estimate of proliferating cardiomyocytes as determined by Ki67 staining in the peri-infarct region 30 days after myocardial infarction. PBS group: *n* = 940 cardiomyocytes from 3 hearts; EV-iPSC group: *n* = 950 cardiomyocytes from 3 hearts; EV-CPC^ISX-9^ group, *n* = 951 cardiomyocytes from 3 hearts. ∗ vs. the PBS group, *P* < 0.05; # vs. the EV-iPSC group, *P* < 0.05. (c) Representative images of arteriole density in the peri-infarct area 4 weeks after MI. Arterioles were identified by *α*-SMA-positive staining (green) of vascular structures. Bar = 100 *μ*m. (d) Quantitative analysis of arteriole density in different treatment groups. ∗ vs. the PBS group, *P* < 0.05; # vs. the EV-iPSC group, *P* < 0.05, *n* = 3.

**Figure 5:**
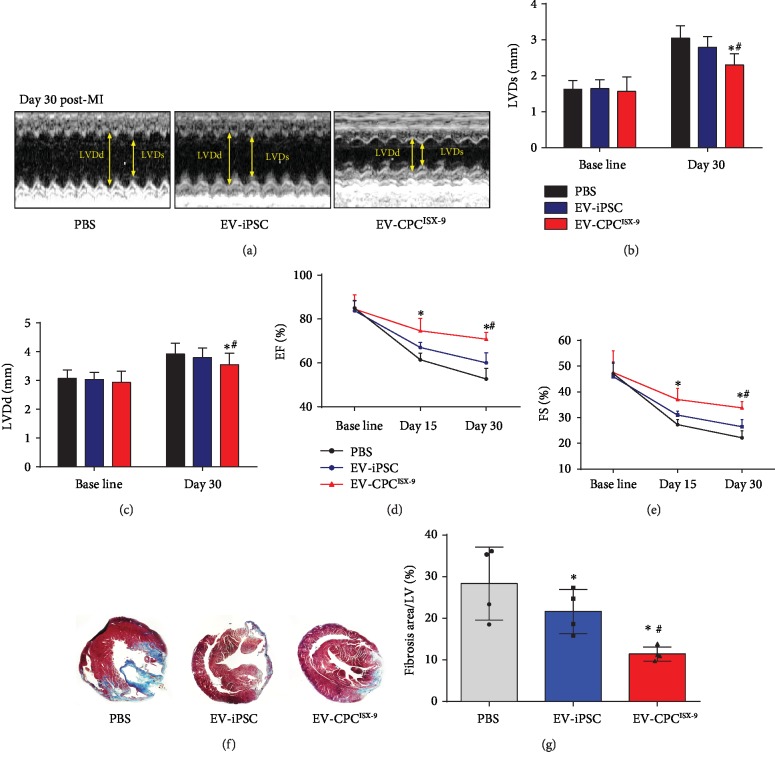
CPC^ISX-9^-derived EV reversed cardiac remodeling in infarcted mice. (a) Representative M-mode echocardiography images from three groups 30 days after MI. LVDs (b), LVDd (c), EF (d), and FS (e) are shown. ∗ vs. the PBS group, *P* < 0.05; # vs. the EV-iPSC group, *P* < 0.05. PBS group: *n* = 10, EV-iPSC group: *n* = 9, and EV-CPC^ISX-9^ group: *n* = 11. EF: ejection fraction; FS: fractional shortening; LVDd: diastolic left ventricular dimensions; LVDs: systolic left ventricular dimensions. (f) Representative Masson's trichrome-stained sections of hearts from the three groups. (g) Quantitative estimate of fibrosis. ∗ vs. the PBS group, *P* < 0.05; # vs. the EV-iPSC group, *P* < 0.05, *n* = 4.

Next, we determined the effects of treatment with EV-CPC^ISX-9^ in a mouse model of MI. Compared to PBS and EV-hiPSCs, EV-CPC^ISX-9^ treatment boosted cardiomyocyte proliferation in the infarcted hearts. Figures 4(a) and 4(b) show representative images and quantitative data of proliferating Ki67 and *α*-actinin positive cardiomyocytes in the peri-infarct region. We further determined the impact of EV-CPC^ISX-9^ on angiogenesis using the tube formation assay. We found that EV-CPC^ISX-9^ indeed increased the average tube length of human aortic endothelial cells (HAECs) in vitro ([Supplementary-material supplementary-material-1]). Remarkably, EV-CPC^ISX-9^ also reduced oxidant-induced changes in HAECs ([Supplementary-material supplementary-material-1]). Similarly, the vessel density as identified by *α*-SMA staining and tube-like structures (Figures 4(c) and 4(d)) in the infarcted region was also increased by treatment with EV-CPC^ISX-9^. The deterioration in cardiac function, as noted by the rise in LVEDD and LVESD as well as a progressive decline in LVFS 1-month post-MI, was attenuated by EV-CPC^ISX-9^ treatment. EV-CPC^ISX-9^ slowed the progression of left ventricle enlargement (LVDs, 2.35 ± 0.31 mm vs. 2.79 ± 0.30 mm and 3.047 ± 0.35 mm; LVDd, 3.54 ± 0.40 mm vs. 3.79 ± 0.33 mm and 3.91 ± 0.38 mm in EV-hiPSC-, PBS-, and EV-CPC^ISX-9^-treated groups, respectively) and improved cardiac function (LVFS: 33.77 ± 2.42% vs. 26.44 ± 2.79% and 22.16 ± 2.78% in EV-hiPSC-, PBS-, and EV-CPC^ISX-9^-treated groups, respectively; LVEF: 70.82 ± 3.12% vs. 52.67 ± 4.78%, and 60.05 ± 4.58% in EV-hiPSC- and PBS-treated groups) (Figure 5(a)–5(e)). Moreover, smaller scar size was observed in mice treated with EV-CPC^ISX-9^ compared with those treated with PBS and EV-hiPSC (*P* < 0.01; Figures 5(f) and 5(g)).

### 3.5. miR-373 Mimic Attenuated Cardiac Fibrosis and Improved Cardiac Function and Angiogenesis after MI

**Figure 6:**
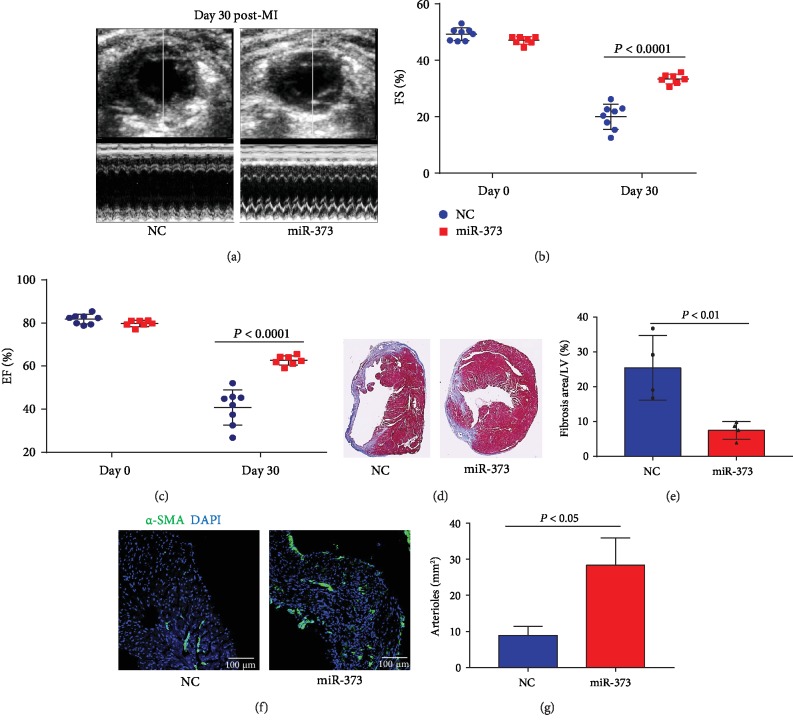
miR-373 mimic improved cardiac function and angiogenesis and attenuated cardiac fibrosis after MI. (a) Representative M-mode echocardiography images from miRNA mimic negative control- (NC-) treated mice and miR-373 mimic-treated mice 30 days post-MI. FS (b) and EF (c) are shown; *P* < 0.001, *n* = 8 in the NC group and *n* = 7 in the miR-373 mimic group. (d) Representative Masson's trichrome-stained sections of hearts from NC-treated mice and miR-373 mimic mice. (e) Quantitative analysis of fibrosis post-MI. (f) Vessel density was assessed by *α*-SMA-positive staining (green) of vascular structures. Bar = 100 *μ*m. (g) Quantitative estimate of arteriole density. *P* < 0.05. *n* = 3 in each group. Schematic depiction of mechanisms of protection by EV-CPC^ISX-9^: role of miR-373 in suppressing fibrosis by targeting two genes, GDF-11 and ROCK-2, and inhibiting myofibroblast differentiation. Myocyte proliferation and angiogenesis were also promoted by EV-CPC^ISX-9^.

Having demonstrated the antifibrotic effects of miR-373 in vitro, we further explored and validated direct effects of miR-373 on postinfarct remodeling and fibrosis. We delivered miR-373 mimic by intramyocardial injection after LAD ligation. After 1 month, miR-373 mimic treatment significantly improved cardiac function compared to control mice (LVFS: 33.38 ± 1.72% vs. 19.98 ± 4.45% in NC-treated mice; LVEF: 62.25 ± 2.16% vs. 40.87 ± 8.17% in NC-treated mice; Figure 6(a)–6(c)). In addition, the miR-373 mimic dramatically attenuated cardiac fibrosis in comparison to NC treatment (Figure 6(d)–6(e) and [Supplementary-material supplementary-material-1]). Moreover, the vessel density as identified by *α*-SMA staining and tube-like structures (Figures 6(f) and 6(g)) in the infarcted region was increased by miR-373 treatment. Despite the effectiveness of EV-CPC^ISX-9^ or miR-373, no difference in the survival rate between the two groups was observed due to death because of cardiac rupture (Figures [Supplementary-material supplementary-material-1]).

## 4. Discussion

Stem cell-based therapy has been well recognized to improve cardiac function following MI. While this therapy has merits, it also suffers from several limitations, particularly lack of suitable stem cell type and their insufficient engraftment and growth, ranging from no new cell formation to sparse newly formed cells in the infarcted tissue [18–20]. Cellular therapy has been propelled by the invention of iPS cells, which have the ability to transform into different progenitor cell types. The cardiac progenitors derived from iPS cells and their counter parts have been used both in animal models of MI [21, 22] and in humans [23] with promising results. While the underlying mechanisms of beneficial effects of stem cell therapy remain a point of debate, increasing evidence suggests that paracrine factors play a key role by reducing cell death and stimulating cell migration and proliferation [24, 25]. This paracrine signaling involves the secretion of small vesicles or EV harboring multiple miRNAs, proteins, and other factors that mediate protection in the heart. Secreted extracellular vesicles (EV) are packed with potent prorepair proteins and RNA cargo that are both cell type-specific and differentially produced and secreted according to the cellular environment. Additionally, miRNA profiles of EV might be distinct from cellular miRNA patterns [26].

In this study, EV derived from CPC^ISX-9^ were found to be highly cardioprotective, and the effect can in part be attributed to their specific miRNA content. CPC^ISX-9^-derived EV were highly enriched with miR-520/-373 family members including miR-371, miR-372, miR-373, and miR-520, as well as miR-512, miR-548, and miR-367, compared to EV derived from other parent cells. miR-373, which was particularly highly enriched in EV-CPC^ISX-9^, was first identified as a human embryonic stem cell- (ESC-) specific miRNA, implicated in the regulation of cell proliferation, apoptosis, senescence, migration, and invasion, as well as DNA damage repair following hypoxia stress [27].

Little has been published regarding the putative role of miR-373 in regulating cardiac pathology or function. In a mouse model of type 1 diabetic cardiomyopathy, miR-373 was found to be significantly downregulated, and application of a miR-373 mimic to neonatal cardiomyocytes exposed to elevated glucose in vitro suppressed cell hypertrophy [28]. Fibrosis is also an important pathological feature of diabetic cardiomyopathy, but effects of miR-373 on fibrosis were not investigated in that study. Fibrosis plays a prominent role in ventricular remodeling and ultimately in the pathogenesis of heart failure after MI. A previous study reported that miR-373 targeted the members of TGF-*β* signaling including TGF-*β* receptor 2 and Smad2 and promoted mesoderm differentiation in human embryonic stem cells [17]. miR-373-3p expression was low in the hypertrophic myocardium with diffuse myocardial fibrosis [29], suggesting that miR-373 may function as an antifibrotic miRNA. Thus, we hypothesized that because of their enrichment in miR-373, EV-CPC^ISX-9^ might produce strong antifibrotic effects to modulate cardiac remodeling.

Our results indicate that both EV-CPC^ISX-9^ and the miR-373 mimic inhibited TGF-*β*- and hypoxia-induced fibrotic gene expression in vitro. With inhibition of miR-373 in EV-CPC^ISX-9^, or treatment with miR-373 inhibitor, the effects on fibrotic gene expression were abrogated. The luciferase activity assay confirmed that miR-373 targeted GDF-11 and ROCK-2, both known to be involved in fibrosis. An isoform of Rho-associated coiled-coil forming protein kinase 2, ROCK-2 is reportedly a critical mediator of organ fibrosis. Inhibition of ROCK-2 protected ROCK-2-haploinsufficient mice from bleomycin-induced myofibroblast differentiation and pulmonary fibrosis [30], while its activation was implicated in the development of idiopathic pulmonary fibrosis [31]. Additionally, fibroblast-specific ROCK-2 was reported to promote cardiac hypertrophy, fibrosis, and diastolic dysfunction due to upregulation of the profibrotic gene (CTGF) and promyofibroblast differentiation (*α*-SMA) genes [32]. Mutant mice with elevated fibroblast ROCK activity exhibited enhanced Ang II-stimulated cardiac hypertrophy and fibrosis [32]. The role of the second identified target gene, GDF-11, is more controversial. It was reported to beneficially reverse age-related cardiac hypertrophy and skeletal muscle dysfunction [33, 34], while other reports suggest that it promotes cardiac and skeletal muscle dysfunction and wasting [35], inhibits skeletal muscle regeneration [36], exerts profibrotic effects [37], and promotes renal failure and interstitial fibrosis [38]. Therefore, our data suggest that miR-373 inhibited profibrotic gene upregulation and myofibroblast differentiation in fibroblasts by targeting GDF-11 and ROCK-2.

In vivo data showed that compared with EV-hiPSC and PBS, EV-CPC^ISX-9^ treatment reduced fibrosis and improved cardiac function, thus supporting a therapeutic role for EV-CPC^ISX-9^ in cardiac remodeling. Given that EV-CPC^ISX-9^ were found to be highly enriched in miR-373, we tested the effects of EV-CPC^ISX-9^ injection in the heart and miR-373 mimic treatment in vivo on the miR-373 expression level and found that the miR-373 expression level in the heart was increased ([Supplementary-material supplementary-material-1]) and it significantly decreased fibrosis and improved cardiac function post-MI. Moreover, the miR-373 mimic also promoted angiogenesis, which was likely mediated by its ability to activate HIF downstream signaling [39]. These findings suggest that the antifibrotic effects of EV-CPC^ISX-9^ are, at least in part, mediated by miR-373, and they also support the notion that the miR-373 mimic might represent a novel therapeutic strategy for controlling fibrosis and cardiac remodeling post infarction and perhaps in other disorders, such as diabetic cardiomyopathy.

The second major effect of EV-CPC^ISX-9^ was on cardiomyocyte proliferation in the infarcted myocardium. A previous study reported that miR-294 (miR-290 cluster), the mouse homolog of human miR-371/372/373 cluster, had a strong effect on cardiac progenitor cell proliferation [40] and that its overexpression led to differentiation towards the mesendoderm lineage [17]. It should be borne in mind, however, that these effects could also be attributed to other miRNAs present in the EV, including miR-302, miR-548, miR-512, and miR-367. Further studies are required to dissect the role of individual EV-CPC^ISX-9^ miRNAs in regulating cardiac fibrosis, cardiomyocyte proliferation, and other pathological events in the context of postinfarction remodeling.

## 5. Conclusion

Several clinical and investigational reports have demonstrated the therapeutic applications of cardiac progenitor cells for the treatment of the ischemic heart. Consequently, these studies led to advance new cell-free (EV) strategies to overcome the limitations of cell-based approaches with the same effectiveness and outcomes. The intracoronary administration of EV eliminates the need for open heart surgery for intramyocardial administration of stem cells. However, the promise of EV does not establish the fact whether their effect is continuous and permanent or if future efforts should continue on strategies directed towards successful engraftment and survival of iPSC-derived cardiac progenitors as a source for new myofiber growth and EV for paracrine effects as well.

**Figure 7:**
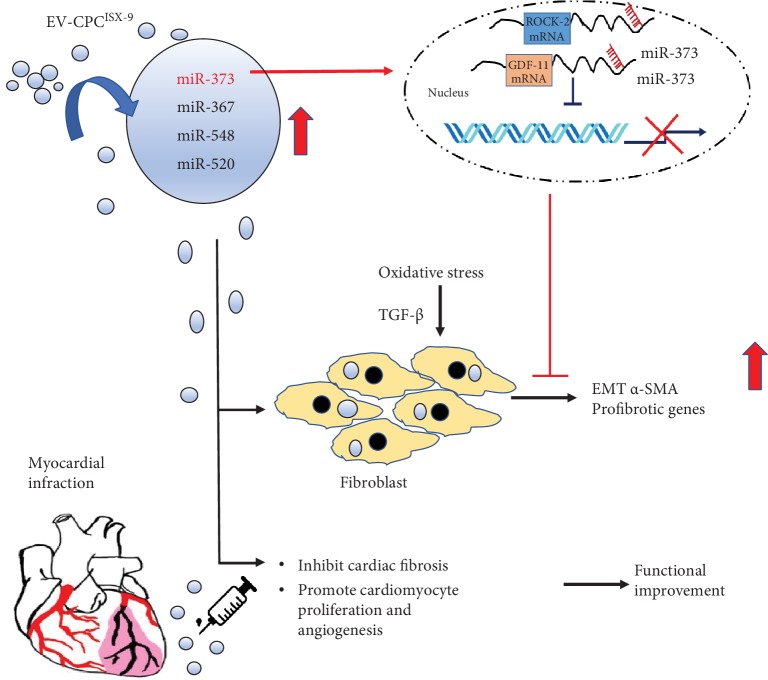
Schematic depiction of mechanisms of protection by EV-CPC^ISX-9^: role of miR-373 in suppressing fibrosis by targeting two genes, GDF-11 and ROCK-2, and inhibiting myofibroblast differentiation. Myocyte proliferation and angiogenesis were also promoted by EV-CPC^ISX-9^.

In summary (Figure 7), we report that EV-CPC^ISX-9^ exhibit a unique miRNA profile and that miR-373 is particularly highly enriched in these EV. EV-CPC^ISX-9^ elicit strong antifibrotic effects, which is attributed at least in part to their enrichment in miR-373. Treatment with EV-CPC^ISX-9^ in vivo reduced postinfarction fibrosis and remodeling, promoted cardiomyocyte proliferation and angiogenesis, and improved cardiac function, findings which were at least in part recapitulated by direct application of miR-373 mimic. These findings have important implications for understanding the paracrine mechanisms of stem cell function and advancing the field of cardiac stem cell therapeutics.

## Data Availability

The raw data of miRNA array is deposited in the GEO database (GSE126347). Other data are available from the authors upon reasonable request.

## Supplementary Materials

Supplementary Materials Figure S1: extracellular vesicle (EV) internalization in fibroblasts. PKH26-labeled EV from CPC^ISX-9^ (red) were observed inside the fibroblasts (green, Calcein AM), mostly located at the perinuclear region. The white arrows indicated the uptake of EVs. Bar = 100 *μ*m. Figure S2: miR-373 expression of CPC^ISX-9^ and their EV. The expression of miR-373 in EV was much higher than in their donor cells. Figure S3: miR-373 inhibition in CPCsISX-9 and their EV by miR-373 inhibitor. (A) Real-time PCR result showed that both 25 nM and 50 nM anti-miR-373 (miR-373 inhibitor) effectively downregulated miR-373 expression in CPC^ISX-9^. (B) Expression of EV miR-373 was significantly inhibited from CPC^ISX-9^ with 25 nM anti-miR-373 treatment. (C) miR-373 expression in fibroblasts with EV-CPC^ISX-9^ or EV-CPC^ISX-9^+anti-miR-373 treatment. Data were from three replicated independent experiments, *n* = 3, *P* < 0.001. Figure S4: human miR-373 3′-UTR binding sites of GDF-11 and ROCK-2 are conserved among species including mice and rat. Conserved sites for miR-373 are found in the ROCK-2 3′-UTR (A) and GDF-11 3′-UTR (B). Figure S5: the effect of EV-CPC^ISX-9^ on tube formation in vitro. (A) Representative images of tube formation in human aortic endothelial cells (HAECs) with EV-CPC^ISX-9^ treatment (1 *μ*g/well, 24-well plate). Cells were labeled with Calcein AM. Bar = 500 *μ*m. (B) EV-CPC^ISX-9^ significantly promoted tube formation of HAECs with increased tube length. Figure S6: EV-CPC^ISX-9^ prevented H_2_O_2_-induced oxidative stress. (A) Representative images of dihydrorhodamine 123 (DHR 123) and MitoTracker staining in human aortic endothelial cells (HAECs) with 100 *μ*M H_2_O_2_ treatment for 8 h. (B) Quantification plot for DHR 123 fluorescence intensity. Figure S7: Masson's trichrome-stained sections in miR-373 mimic-treated mice 30 days post-MI. Representative Masson's trichrome images of LV sections show a thicker scar in NC-treated mice compared to miR-373 mimic-treated mice. Bar = 100 *μ*m. Figure S8: survival of miR-373 mimic-treated mice after MI. (A) Survival analysis showed no significant difference between miR-373 mimic-treated mice and negative control-treated mice. *n* = 12 in the NC group; *n* = 10 in the miR-373 mimic group. (B) Autopsy photo from a dead mouse. Hemorrhage (blood clots) in the chest and obvious rupture slit in the left ventricle free wall were identified. Figure S9: survival analysis in EV-treated mice after MI. Survival analysis showed no significance was found among PBS-, EV-iPSC-, and EV-CPC^ISX-9^-treated mice. *n* = 13 in each group. Figure S10: miR-373 expression in mouse heart 24 h after EV-CPC^ISX-9^ intramyocardium injection. miR-373 expression was increased in the heart tissue after EV-CPC^ISX-9^ injection.
