# Supplementary material for: miRNAs in Extracellular Vesicles from iPS-Derived Cardiac Progenitor Cells Effectively Reduce Fibrosis and Promote Angiogenesis in Infarcted Heart
Source: Stem Cells Int. 2019 Nov 11;2019:3726392. doi: 10.1155/2019/3726392 (PMC6878789; doi:10.1155/2019/3726392)
Supplement: Supplementary Materials — Figure S1: extracellular vesicle (EV) internalization in fibroblasts. PKH26-labeled EV from CPCISX-9 (red) were observed inside the fibroblasts (green, Calcein AM), mostly located at the perinuclear region. The white arrows indicated the uptake of EVs. Bar = 100 μm. Figure S2: miR-373 expression of CPCISX-9 and their EV. The expression of miR-373 in EV was much higher than in their donor cells. Figure S3: miR-373 inhibition in CPCsISX-9 and their EV by miR-373 inhibitor. (A) Real-time PCR result showed that both 25 nM and 50 nM anti-miR-373 (miR-373 inhibitor) effectively downregulated miR-373 expression in CPCISX-9. (B) Expression of EV miR-373 was significantly inhibited from CPCISX-9 with 25 nM anti-miR-373 treatment. (C) miR-373 expression in fibroblasts with EV-CPCISX-9 or EV-CPCISX-9+anti-miR-373 treatment. Data were from three replicated independent experiments, n = 3, P < 0.001. Figure S4: human miR-373 3′-UTR binding sites of GDF-11 and ROCK-2 are conserved among species including mice and rat. Conserved sites for miR-373 are found in the ROCK-2 3′-UTR (A) and GDF-11 3′-UTR (B). Figure S5: the effect of EV-CPCISX-9 on tube formation in vitro. (A) Representative images of tube formation in human aortic endothelial cells (HAECs) with EV-CPCISX-9 treatment (1 μg/well, 24-well plate). Cells were labeled with Calcein AM. Bar = 500 μm. (B) EV-CPCISX-9 significantly promoted tube formation of HAECs with increased tube length. Figure S6: EV-CPCISX-9 prevented H2O2-induced oxidative stress. (A) Representative images of dihydrorhodamine 123 (DHR 123) and MitoTracker staining in human aortic endothelial cells (HAECs) with 100 μM H2O2 treatment for 8 h. (B) Quantification plot for DHR 123 fluorescence intensity. Figure S7: Masson's trichrome-stained sections in miR-373 mimic-treated mice 30 days post-MI. Representative Masson's trichrome images of LV sections show a thicker scar in NC-treated mice compared to miR-373 mimic-treated mice. Bar = 100 μm. Figure S8: surviva [file 3726392.f1.pdf]

Supplemental Methods

Cell culture

Human iPSC cell line (ACS-1021, ATCC, USA) was maintained in mTeSR1 media (Stem Cell Technology) on vitronectin coated six-well plates. Cells were passaged with ReLeSR™ reagent every 4-7 days according to the manufacturer's protocol (Stem Cell Technology). For CPC generation, briefly, hiPSCs maintained on vitronectin coated six-well plates in mTeSR1 media (Stem Cell Technology) were dissociated into single cells using accutase (Invitrogen) at 37℃ for 10 min and then were seeded on to a vitronectin-coated six-well plates at 1×10^6 cells/well in mTeSR1 supplemented with 5 µM ROCK inhibitor (Y-27632, Stem Cell Technology) for 24 h. The following day, cells were cultured in mTesR1 with daily medium change for 3 days. Afterwards, the medium was switched to RPMI/B27 minus insulin supplemented with ISX-9 (20 µM, dissolved in DMSO, Stem Cell Technology) for 7 days. Embryoid bodies (EB) were generated using the hanging drop method in RPMI/B27 minus insulin medium. Human dermal fibroblast cell line (CC-2511) and lung fibroblast cell line (CC-2512) were obtained from Lonza Company. Briefly, fibroblasts were maintained in FibroGRO™ Complete Media (Millipore Sigma). Cells were passaged with accutase; passages 2-4 were used for experiments. Commercial human CPCs derived from human iPS cells (Catalog: R1093, Cellular Dynamics International) were maintained in serum-free William's E Medium supplemented with Cocktail B (CM400, Life Technologies). Passage 2 was used for experiments.

miRNA Array analysis

The assay allows measurement of 800 different microRNAs at the same time for each sample. 3.5 µl of suspension RNA was annealed with multiplexed DNA tags (miR-tag) and bridges target specifics. Mature microRNAs were bonded to specific miR-tags using a Ligase enzyme, and excess tags were removed by enzyme clean-up step. The tagged microRNA product was diluted 1 to 5, and 5 µl was combined with 20 µl of reporter probes in hybridization buffer and 5 µl of Capture probes overnight (17 hours) at 65°C to permit hybridization of probes with specific target sequences. Excess probes were removed using two-step magnetic bead-based purification on an automated fluidic handling system (nCounter Prep Station) and target/probe complexes were immobilized on the cartridge for data collection. The nCounter Digital Analyzer took images of immobilized fluorescent reporters in the sample cartridge with a CCD camera through a microscope objective lens. For each cartridge, a high-density scan encompassing 325 fields of view was performed. NanoString raw data was analyzed with nSolver™ software, provided by NanoString Technologies. The mean plus 2 times the standard deviation of negative control probes was used to perform background subtraction; positives were used to perform technical normalization to adjust lane by lane variability due to differences in hybridization, purification or binding. Data was then normalized by calculating the geometric mean of the spikes present in each sample, as recommended by NanoString. One-way ANOVA was used to calculate the P value; targets with $P < 0.05$ were selected.

miRNA target gene prediction, gene ontology(GO) analysis and luciferase activity assay

Differentially miRNA target genes in significant GO and pathway categories, obtained from GO and pathway analyses, were analyzed with mirPath v.3 software. GO biological process includes biological processes, molecular function and cellular component of upregulated and downregulated genes.

For luciferase activity assay, using standard procedures, wild-type (WT) or mutant 3'untraslated regions (UTRs) of GDF-11 or ROCK-2 were subcloned into the pLenti-UTR-Dual–Luc vector (abm, Canada) (Fig.3C) downstream of the luciferase gene. The predicted binding sites and mutant sequences are shown in Fig.3D. GDF-11-3'UTR-WT, GDF-11-3'UTR-Mut, ROCK2-3'UTR-WT, or ROCK2-3'UTR-Mut vectors were co-transfected with miR-373 mimic or negative control into 293FT cells using Lipofectamine 3000 for 48 h. Transfected cells were analyzed using the dual-luciferase reporter assay system (Promega). The luciferase activity was normalized using Renilla activity.

Echocardiography

Hearts were imaged in 2D in the parasternal long-axis and/or parasternal short-axis views at the level of the highest LV diameter. Measurements of left ventricular end diastolic diameter (LVDd), and left ventricular end systolic diameter (LVDs) were made from 2D M-mode images of the left ventricle in both systole and diastole. Left ventricle fractional shortening (LVFS) was calculated using the following formula: LVFS= (LVDd-LVDs)/LVDd x 100. Ejection fraction (EF), Left ventricular end diastolic volume (LVEDv) and left ventricular end systolic volume (LVESv) were calculated using the following formula: $7.0 \times LVEDd^3/\ (2.4 + LVDd)$ and $7.0 \times LVESd^3/\ (2.4 + LVDs)$ respectively; left ventricular ejection fraction (LVEF) was calculated as (LVEDv - LVESv)/LVEDv × 100%. LVFS and EF were expressed as percentages.

Histology

For immunostaining, hearts were fixed with 4% PFA for 1 hour at room temperature and replaced by 30% sucrose overnight at 4°C. Afterwards, hearts were cryopreserved in an optical cutting temperature (OCT) compound (Tissue Tek) at -80°C. Hearts were sliced into 5-μm-thick frozen sections and incubated with primary antibodies including α-sarcomeric actinin (A7811, Sigma, 1:200), ki67 (ab16667, abcam, 1:500), cTnT (13-11, Thermo fisher Scientific, 1:300) and SMA (ab5694, abcam, 1:300). Signals were visualized with Alexa Fluor 647 and Alexa Fluor 488 secondary antibodies (Life Technologies). Images were recorded on a confocal microscope (FV1000, Olympus, Japan). For fibrosis analysis, hearts were embedded in paraffin and cut at 5-μm-thick sections. Masson trichrome staining was performed according to the manufacturer's protocol (HT-15, Sigma). The size of LV area and scar area were measured using the ImageJ software. 4 sections (EV treated mice) and 6 sections (miR-373 mimic treated mice) were analysed per heart. The fibrosis area was determined as the ratio of scar area to LV area and expressed as percentage. Vessel density was determined in 9 animals (3 in each group) in NOD/SCID mice, and 6 animals in C57/B6 mice (3 in each group) which were sacrificed at 1M after MI. The number of vessels was blindly counted on 27 sections (3 sections per heart) in NOD/SCID mice or 18 sections (3 sections per heart) in C57/B6 mice in the infarct and border areas of all mice after staining with an antibody α-SMA using a fluorescence microscope at a 400 x magnification. Vascular density was determined by counting α-SMA positive vascular structures. The number of vessels in each section was averaged and expressed as the number of vessels per field (0.2 mm^2).

Western blot

10 μg proteins were separated by SDS/PAGE and transferred to PVDF membrane (BioRad). Membranes were incubated with primary antibodies against the following proteins overnight at 4°C: mouse anti-tsg101 (sc-365062, Santa Cruz), mouse anti-Calnexin (sc-23954, Santa Cruz), goat-anti-Hsp70 (EXOAB-Hsp70A-1, SBI), rabbit anti-CD9 (#13174, CST), rabbit anti-Flotillin-1(#18634, CST), mouse anti-GADPH (sc-365062, Santa Cruz). The membrane was then washed, incubated with an anti-mouse/rabbit/goat peroxidase-conjugated secondary antibody. Immunoreactive bands were visualized by the enhanced chemiluminescence method (Pierce, Thermo Scientific) with a western blotting detection system (Fluorchem E, ProteinSimple USA) and were quantified by densitometry with ImageJ software.

RNA extraction and real time PCR

Quantification of mRNA and selected miRNAs were performed by real-time system quantstudio3 (ABI) using miScript SYBR Green PCR Kit (Qiagen). miRNA primer sequences are shown in Table S1, and mRNA primer sequences are shown in Table S2. Expression levels of selected miRNAs were quantified, validated with RT-PCR and values are expressed as $2^{-\Delta\Delta CT}$ with respect to the expression of the reference U6. The primer of U6 was provided in the PCR kit.

Tube formation Assay

Human aortic endothelia cell (HAEC, 1×10^5 cells/well) was seeded on Matrigel (Corning) in a 24-well plate and treated with or without 1 μg EV-CPC^{ISX-9} in EGM-2V basal medium (Lonza). After 16 h, cells in Matrigel were stained with Calcein AM, and images were taken by fluorescent microscope. Average tube length was analyzed by Image J software.

Reactive oxygen species (ROS) measurement

HAEC were pretreated with EV free culture medium with either PBS or 1 μg EV-CPC^{ISX-9} for 24h and then cells were subjected to 100 μM H_2O_2 for 8h. Cells were stained with dihydrorhodamine 123 (20 μM) and Mitotracker orange and reactive oxygen species (ROS) production was visualized with fluorescence microscope. Fluorescence intensity was analyzed using Image J software. Results are calculated as a percentage of fluorescence intensity compared with the PBS treated group.

Supplemental Figures

Fig.S1 Extracellular vesicle (EV) internalization in fibroblasts. PKH 26 labeled EV from CPC^{ISX-}9 (red) were observed inside the fibroblasts (green, Calcein AM), mostly located at the perinuclear region. The white arrows indicated the uptake of EVs. Bar = 100 μm.

Fig.S2 miR-373 expression of CPC^{ISX-9} and their EV. The expression of miR-373 in EV was much higher than in their donor cells.

Fig. S3 miR-373 inhibition in CPCs^{ISX-9} and their EV by miR-373 inhibitor. (A) Real-time PCR result showed both 25 nM and 50 nM anti-miR-373 (miR-373 inhibitor) effectively downregulated miR-373 expression in CPC^{ISX-9}. (B) Expression of EV miR-373 was significantly inhibited from CPC^{ISX-9} with 25

nM anti-miR-373 treatment. (C) miR-373 expression in fibroblasts with EV-CPC^{ISX-9} or EV-CPC$^{ISX-9+anti-miR-373}$ treatment. Data were from three replicated independent experiments, n = 3, $P < 0.001$.

Fig. S4 human miR-373 3' UTR binding sites of GDF-11 and ROCK-2 are conserved among species including mice and rat. Conserved sites for miR-373 are found in the ROCK-2 3-UTR (A) and GDF-11 3-UTR (B).

Fig.S5 The effect of EV-CPC^{ISX-9} on tube formation in vitro. (A) Representative images of tube formation in human aortic endothelia cells (HAECs) with EV-CPC^{ISX-9} treatment (1 µg/well, 24 well plate). Cells were labeled with Calcein AM. Bar=500 µm. (B) EV-CPC^{ISX-9} significantly promoted tube formation of HAEC with increased tube length.

Fig.S6 EV-CPC^{ISX-9} prevented H_2O_2 induced oxidative stress. (A) Representative images of Dihydrorhodamine 123 (DHR 123) and Mitotracker staining in human aortic endothelia cells (HAEC) with 100 µM H_2O_2 treatment for 8h. (B) Quantification plot for DHR 123 fluorescence intensity.

Fig.S7 Masson's trichrome-stained sections in miR-373 mimic treated mice 30 days post-MI. Representative Masson's trichrome images of LV sections show a thicker scar in NC treated mice compared to miR-373 mimic treated mice. Bar = 100 µm.

Fig.S8 Survival of miR-373 mimic treated mice after MI. (A) Survival analysis showed no significant difference between miR-373 mimic treated mice and negative control treated mice. n = 12 in NC group, n = 10 in miR-373 mimic group. (B) Autopsy picture from a dead mouse. Hemorrhage (blood clots) in the chest and obvious rupture slit in the left ventricle free wall were identified.

Fig.S9 Survival analysis in EVs treated mice after MI. Survival analysis showed no significance was found among PBS, EV-iPSC and EV-CPC^{ISX-9} treated mice. n = 13 in each group.

Fig. S10 miR-373 expression in mouse heart 24h after EV-CPC^{ISX-9} intramyocardium injection. miR-373 expression was increased in heart tissue after EV-CPC^{ISX-9} injection.

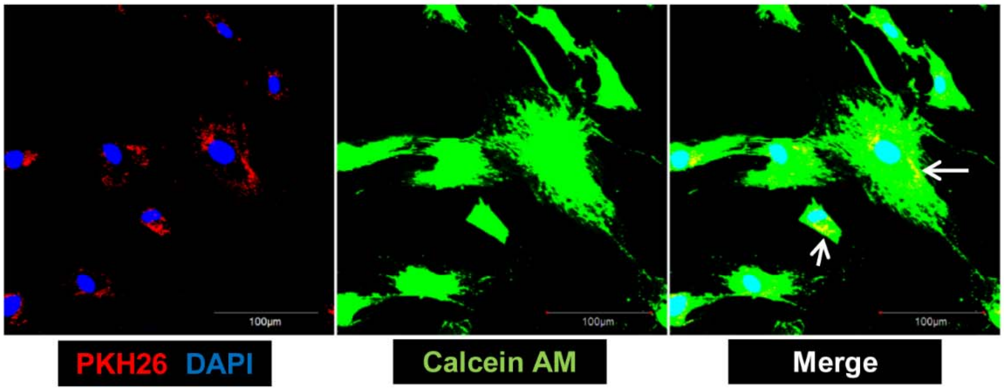
100μm
100μm
100μm
PKH26 DAPI
Calcein AM
Merge

Fig.S1

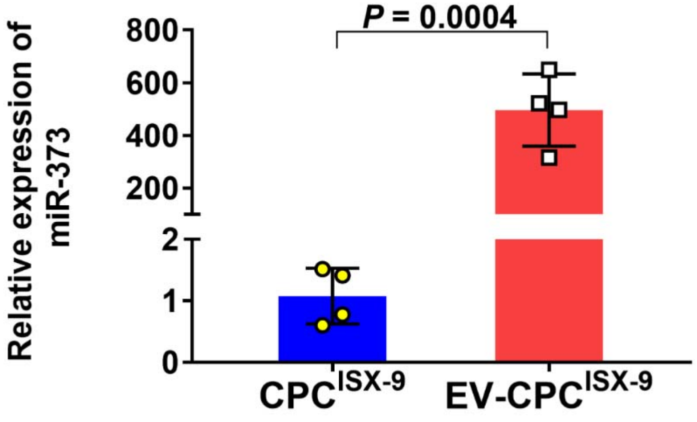
P = 0.0004
Relative expression of miR-373
800
600
400
200
2
1
0
CPC^ISX-9
EV-CPC^ISX-9

Fig.S2

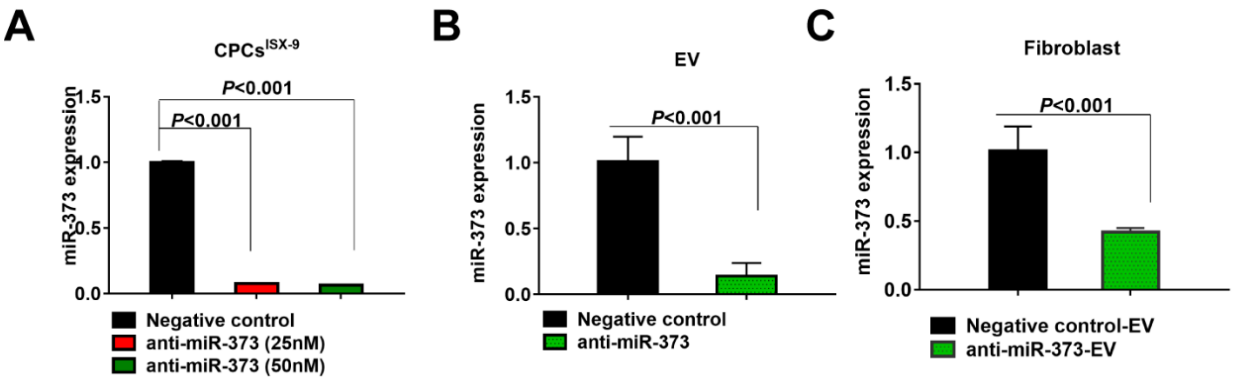

Fig.S3

A

**Human** *ROCK2*	**5'GCTGCAGTTGCAACTATGCACTTG 3'**	**3' UTR (576-583)**
**Mouse** *ROCK2*	**5'ATTTCAGTTGCAACTATGCACTTG 3'**	**3' UTR (571-578)**
**Rat** *ROCK2*	**5' ATTTCAGTTGCAACTATGCACTTG 3'**	**3' UTR (592-599)**
**Horse** *ROCK2*	**5'ACTGCAGTTGCAACTATGCACTTG 3'**	**3' UTR (641-648)**
**Sheep** *ROCK2*	**5' ACTGCAGTTGCAACTATGCACTTG 3'**	**3' UTR (612-619)**
**Chicken** *ROCK2*	**5'ATTGCAGTTGCAACTATGCACTTG 3'**	**3' UTR (569-576)**
	* *********************	
**Hsa-miR-373**	**3' TGTGGGGTTTTAGCTTCGTGAAG 5'**	

B

**Human** *GDF11*	**5' ACACCTACTCACTTAAGCACTTG 3'**	**3' UTR (704-710)**
**Mouse** *GDF11*	**5' ATACCAATCTACTTAAGCACTTG 3'**	**3' UTR (711-718)**
**Rat** *GDF11*	**5' GGTTCCGACCTACTTAAGCACTTG 3'**	**3' UTR (706-713)**
**Horse** *GDF11*	**5'AATACCCACCTTCTTAAGCACTTG 3'**	**3' UTR (740-747)**
**Sheep** *GDF11*	**5' AATACTCACCTACTTAAGCACTTG 3'**	**3' UTR (744-751)**
**Dog** *GDF11*	**5' AATACCCACCTACTTAAGCACTTG 3'**	**3' UTR (779-786)**
**Hsa-miR-373**	**3' TGTGGGGTTTTAGCTTCGTGAAG 5'**	

Fig.S4

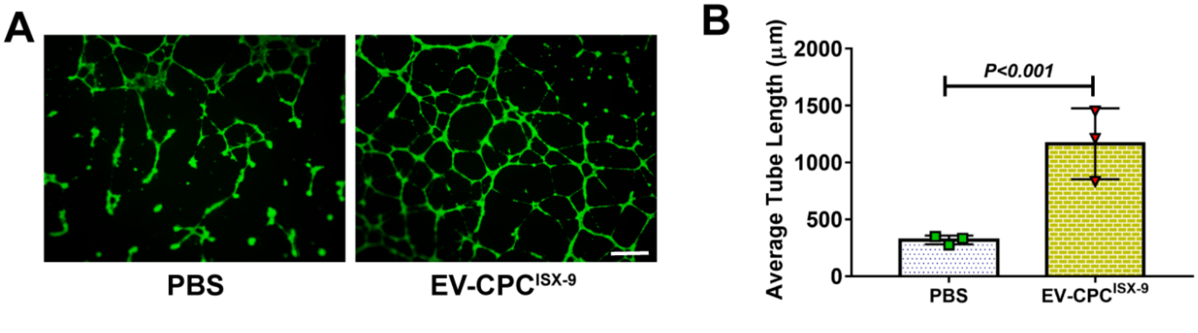
A
PBS
EV-CPC ISX-9
B
Average Tube Length (μm)
2000
1500
1000
500
0
P<0.001
PBS
EV-CPC ISX-9

Fig.S5

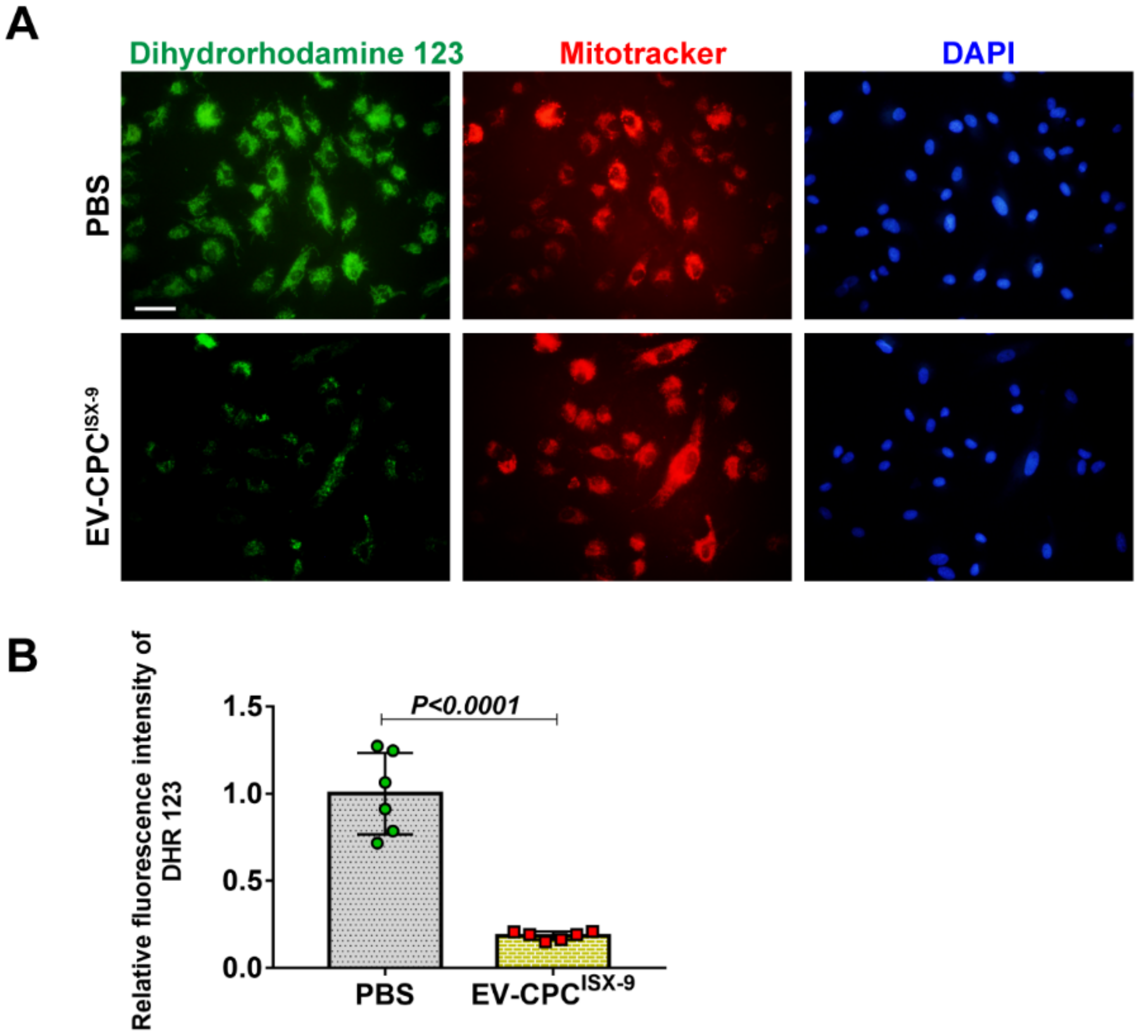
A
Dihydrorhodamine 123
Mitotracker
DAPI
PBS
EV-CPC^ISX-9
B
Relative fluorescence intensity of DHR 123
P<0.0001
1.5
1.0
0.5
0.0
PBS
EV-CPC^ISX-9

Fig.S6

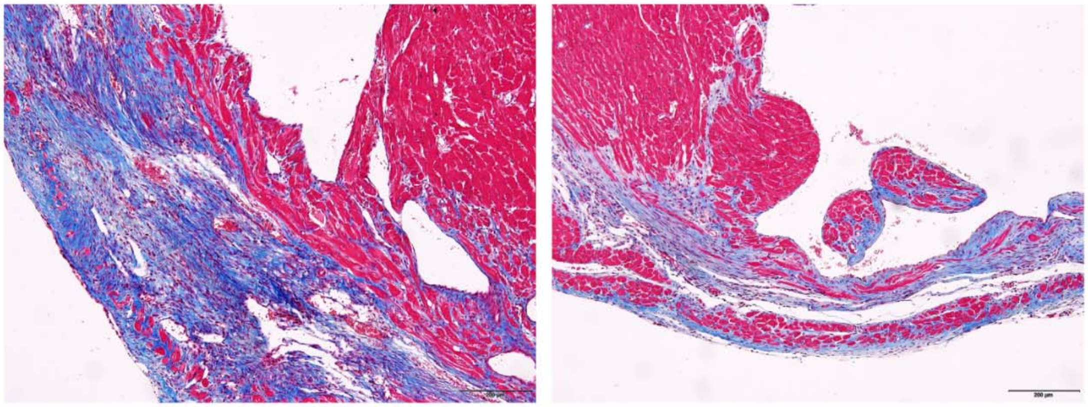

NC miR-373

Fig.S7

A

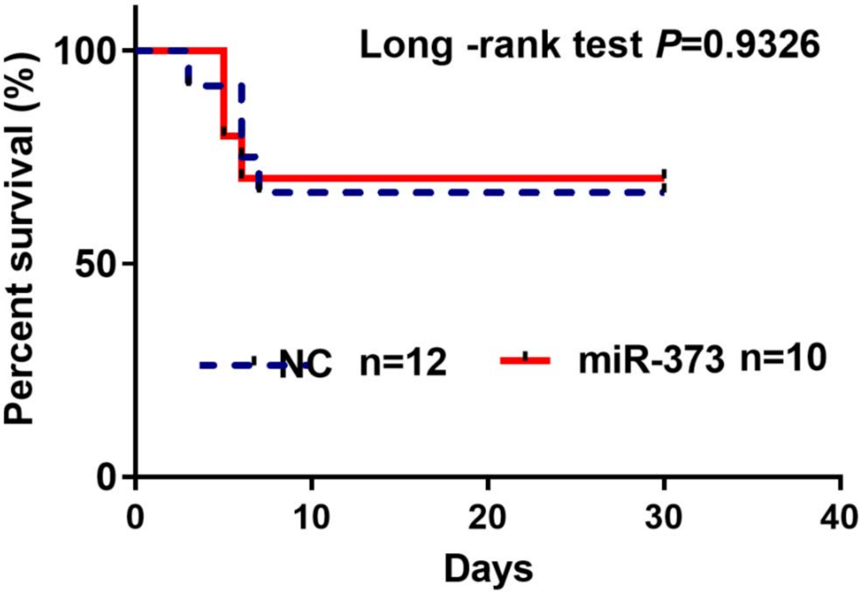

Long -rank test P=0.9326
100
50
0
Percent survival (%)
NC n=12
miR-373 n=10
0
10
20
30
40
Days

B

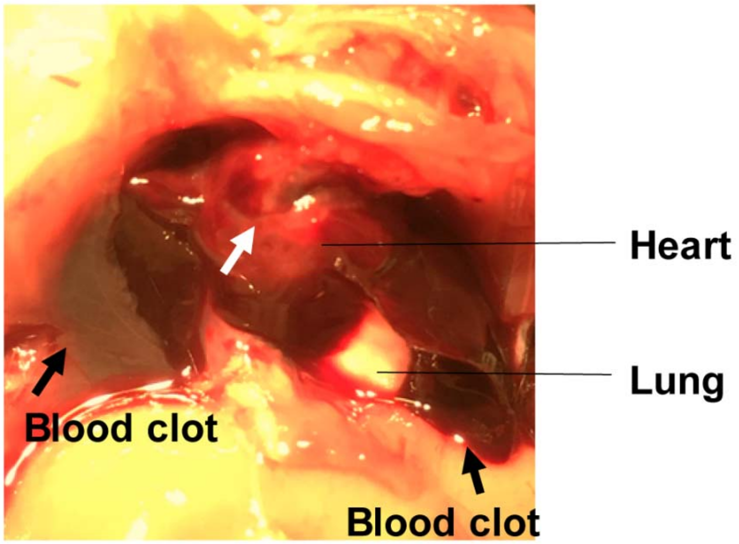

Heart
Lung
Blood clot
Blood clot

Fig.S8

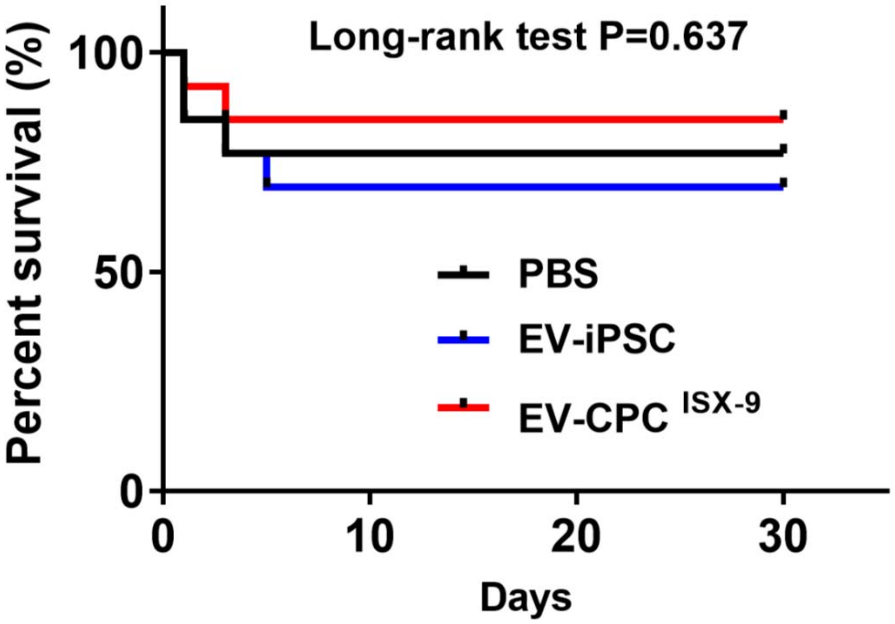

Fig.S9

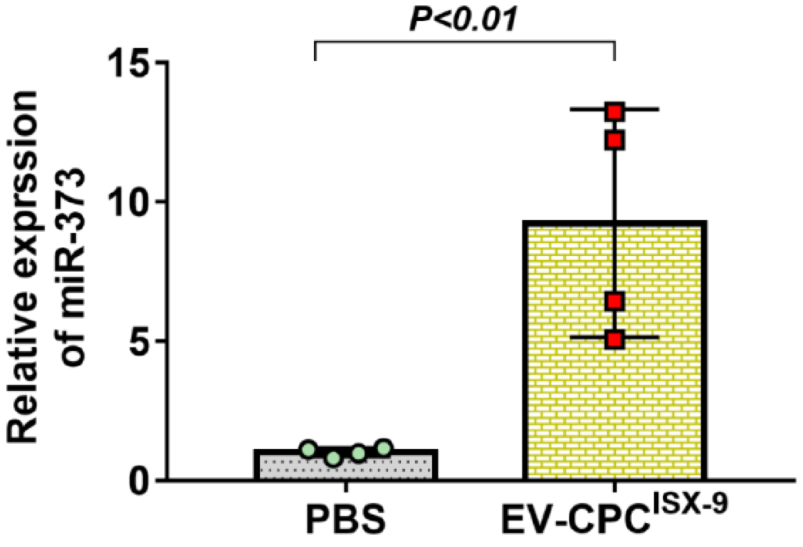

Fig.S10

Supplementary tables

Table S1 Primer sequences of miRNAs

miRNA	Sequence
hsa-miR-373-3p	GAAGTGCTTCGATTTTGGGGTGT
hsa-miR-520a-5p	CTCCAGAGGGAAGTACTTTCT
hsa-miR-367-3p	AATTGCACTTTAGCAATGGTGA
hsa-miR-548ah-5p	AAAAGTGATTGCAGTGTTTG
hsa-miR-548q	GCTGGTGCAAAAGTAATGGCGG

Table S2 Primer sequences of mRNAs

Gene	Forward 5'-3'	Reverse 5'-3'
h-TIMP1	GGAATGCACAGTGTTTCCCTG	GGAAGCCCTTTTCAGAGCCT
h-TIMP2	GCTGCGAGTGCAAGATCACG	TGGTGCCCGTTGATGTTCTT
h-MMP9	AAGGATGGGAAGTACTGGCG	GCTCCTCAAAGACCGAGTCC
h-MMP2	AGATCGCGAGAGAGCAATACC	CTGGGGCAGTCCAAAGAACT
h-CTGF	GAGGAGTGGGTGTGTGACG	TCTTCCAGTCGGTAAGCCGC
h-FN1	ACAAACACTAATGTTAATTGCCCA	CGGGAATCTTCTCTGTCAGCC
h-COL1A1	GGACACAGAGGTTTCAGTGGT	GCACCATCATTTCCACGAGC
h-COL2A1	CATGAGGGCGCGGTAGAG	CCAGCCTCCTGGACATCCT
h-COL3A1	GAAAGATGGCCCAAGGGGTC	TATACCTGGAAGTCCGGGGG
h-GDF-11	ATCGCACCTAAGCGCTACAA	GCTGCACCAAATGGGTATGC
h-ROCK-2	AACGTCAGGATGCAGATGGG	CAGCCAAAGAGTCCCGTTCA
h-GADPH	GAAGACGGGCGGAGAGAAAC	CGACCAAATCCGTTGACTCC
